# Postoperative pain management after VATS for spontaneous pneumothorax - a systematic review

**DOI:** 10.1186/s12871-026-03865-1

**Published:** 2026-05-05

**Authors:** Quirine C. A. van Steenwijk, Loes Janssen, Iris Hoogendoorn, Chris Dickhoff, Jerry Braun, Frank J. C. van den Broek

**Affiliations:** 1 Department of Surgery, Máxima Medical Center, Veldhoven, The Netherlands; 2 Department of Science and Medical Innovation, Máxima Medical Center, Veldhoven, The Netherlands; 3 Department of Cardiothoracic Surgery, Amsterdam UMC, Location VU Medical Center, Cancer Center Amsterdam, Amsterdam, The Netherlands; 4 Department of Cardiothoracic Surgery, Leiden University Medical Center, Leiden, The Netherlands

**Keywords:** Analgesic technique, Spontaneous pneumothorax, Pleurodesis, Pain management, Acute postoperative pain, Thoracic epidural analgesia, Locoregional analgesia, Systemic analgesia

## Abstract

**Background:**

Historically, thoracic epidural analgesia has been the standard for postoperative pain management after surgery for spontaneous pneumothorax. With the advent of enhanced recovery after thoracic surgery protocols, less invasive locoregional analgesic techniques are increasingly considered. Due to lack of high-quality evidence, current guidelines do not address optimal analgesic strategies in this population. This systematic review evaluated the effectiveness of various analgesic techniques following video-assisted thoracoscopic surgery for spontaneous pneumothorax.

**Methods:**

Medline, Embase and Cochrane databases were searched until May 2025. Inclusion criteria were patients undergoing video-assisted thoracoscopic surgery for spontaneous pneumothorax, with clearly described postoperative analgesic management (e.g. epidural, locoregional, systemic) and pain scores (visual analgesic score or numeric pain rating score) within 48 h. The primary outcome was pain scores within 48 h postoperatively; secondary outcomes included adjunct analgesic use and complications. Risk of bias was assessed for the primary outcome.

**Results:**

Thirty studies comprising 3,203 patients were assessed: risk of bias was low in 3, moderate/some concerns in 24 and high in 3 studies. There were 10 comparative studies and 20 single-arm cohort studies applying solely systemic analgesia (*n* = 14) or locoregional analgesia (n = 6). No single-arm cohort study applied solely epidural analgesia. Postoperative pain scores within 48 h were higher after systemic analgesia only, compared to the addition of locoregional or epidural analgesia. No clear differences were observed in analgesia-related complications between the analgesic techniques.

**Conclusions:**

Locoregional or epidural analgesia as part of a multimodal regimen was associated with the lowest postoperative pain scores, although differences were small. Substantial heterogeneity and low certainty of evidence precluded definitive recommendations. This review provides a foundation for future research investigating optimal analgesic strategies following surgery for spontaneous pneumothorax.

**Supplementary Information:**

The online version contains supplementary material available at 10.1186/s12871-026-03865-1.

## Background

Video-assisted thoracoscopic surgery (VATS) with bullectomy and pleurodesis is the treatment of choice for patients with recurrent spontaneous pneumothorax (SP) [1]. Historically, bullectomy and pleurodesis was considered a painful procedure relying on thoracic epidural analgesia (TEA) for postoperative analgesia [2, 3]. The clinically evident pain control by TEA outweighed its side-effects, such as postoperative nausea and vomiting (PONV), pruritus, lower limb weakness and hypotension [3]. Since the implementation of VATS and enhanced recovery after thoracic surgery (ERATS) programs, which both resulted in a reduction of postoperative pain, morbidity and length of hospital stay (LOS), the value of TEA as preferred analgesic approach is under debate [4, 5]. Therefore, less invasive analgesic techniques are being explored. Recent European ERATS guidelines already recommend locoregional analgesia instead of TEA after anatomical lung resections for cancer to enhance early mobilization and reduce the risk of TEA related harms [6]. Several less harmful locoregional analgesic techniques are available, including paravertebral block (PVB), erector spinae plane block (ESPB), serratus anterior plane block (SAPB), and intercostal nerve block (ICNB). Postoperative pain may vary between these techniques due to anatomical differences, as well as due to different possible methods of administration: either as single-shot (ss) injection or continuously via an indwelling perineural catheter for prolonged analgesia [7].

However, due to the lack of high-quality research data on pain management after VATS for SP, the value of locoregional analgesia in these patients remains unclear [6, 7]. In fact, guidelines on the management of SP do not even address postoperative pain management [1, 8]. A recent survey among Dutch thoracic surgeons showed that the preferred analgesic technique after VATS varied between TEA, locoregional analgesia and systemic analgesia [9]. Although TEA was used most, recent cohort studies suggest locoregional techniques to be viable options in these patients [10, 11].

Before implementing the widespread use of locoregional analgesic techniques after VATS for SP, a systematic evaluation of the existing literature regarding pain management is warranted. The aim of this systematic review is to evaluate the effectiveness of locoregional analgesia, TEA and systemic analgesia after VATS for SP. Where possible, two comparisons were performed: (1) regional analgesia (locoregional techniques and TEA) versus systemic analgesia alone, and (2) locoregional analgesia versus TEA.

## Methods

This systematic review was registered in PROSPERO (ID CRD420251048499) on May 11th 2025 and is reported according to the PRISMA reporting checklist (Additional file 1) [12, 13]. Apart from registration in PROSPERO, no additional study protocol was developed.

The definitions used in this systematic review to distinguish between the different types of analgesia were as follows: Locoregional analgesia was defined as all peripheral analgesic techniques other than TEA, which is applied centrally. Regional analgesia encompassed both TEA and/or locoregional techniques. Systemic analgesia was defined as analgesic medication exerting its effect systemically, such as paracetamol, nonsteroidal anti-inflammatory drugs (NSAIDs), or opioids administered orally or intravenously. A multimodal regimen refers to the administration of multiple analgesic agents that may include both systemic analgesics and a nerve block.

### Eligibility criteria

A literature search was conducted using the PICOS framework, focusing on patients undergoing surgery for spontaneous pneumothorax (Participants). The review evaluated systemic and locoregional analgesia for postoperative pain management (Interventions), with comparisons made to regional analgesia or TEA depending on the intervention assessed (Comparison). The primary outcome was postoperative pain within the first 48 h after surgery and secondary outcomes were supplementary analgesic use and complications related to the analgesic technique (Outcomes). Eligible studies comprised clinical randomized and non-randomized controlled trials, as well as observational studies.

Studies were excluded when meeting the following exclusion criteria: surgery by thoracotomy, sample size < 20, absence of reported pain scores, inappropriate study design (e.g. case reports, reviews and editorials), trial registrations, written in a language other than English or indistinct description of analgesic technique. If articles reported on several intervention and control groups from which only a selection was eligible according to our criteria, only the eligible subgroup was included.

### Search strategy and study selection

Studies were identified through an electronic search in Medline (OVID), EMBASE (Embase.com) and Cochrane Library (Wiley) with a time limit from 1995 onwards since from this moment VATS was more readily used [14]. In addition, reference lists of included studies were reviewed for relevant studies (citation tracking). The search strategy was developed by a medical librarian and consisted of a combination of index terms and free text words related to pneumothorax, surgical treatment for pneumothorax and pain management. The final search was conducted on May 19th, 2025. The full search strategy is provided in Additional file 2.

Two reviewers (QvS and LJ) independently screened the titles, abstracts and full-text of all identified studies. Any discrepancies were resolved by consulting the senior author (FvdB).

### Deviations from protocol

Our initial PROSPERO registration indicated postoperative LOS and mobility as secondary outcome measures. However, since the majority of retrieved studies focused on primary outcomes different from pain, the reported LOS was merely determined by factors other than pain management (e.g. surgical or anaesthesiological technique). Moreover, mobility was reported in only one of the retrieved studies. To prevent misleading conclusions, both LOS and mobility were therefore omitted as secondary outcomes in this review.

### Quality assessment

The methodological quality of all included studies was independently assessed by two reviewers (QvS and LJ) and any discrepancy was resolved by discussion and consensus. The risk of bias (RoB) of RCTs was evaluated using the revised Cochrane Risk of Bias tool (RoB 2) [15]. For non-RCTs the risk of bias tool for non-randomized studies for interventions (ROBINS-I) was used [16]. (Non-)RCTs lacking a direct comparison between two pain management strategies were analyzed as single-arm cohort studies, which precluded the assessment of RoB in the confounding domain for non-RCTs and the randomization domain for RCTs (Additional file 3 risk of bias assessment). A study was defined as low risk of bias in case not a single item from the RoB 2 or ROBINS-I tool was scored as moderate or high risk of bias.

### Data extraction

Data was extracted by one author (QvS) using a data extraction form. Extracted information included study details (year, first author, study design and total number of patients included), surgical procedure (access, technique, type of pleurodesis and/or bullectomy), analgesic technique (type, medication, dosage and duration), all pain scores up to 48 h postoperatively, additional analgesia (type, medication, dosage and duration) and postoperative complications related to the analgesic technique (e.g. bleeding at puncture site, PONV, headache, allergic reactions, urinary retention, and hypotension). The exact number of each complication was reported with its respective percentage, using all included patients in the denominator. We sought to determine whether single patients experienced more than one complication. In cases of uncertainty during data extraction, a second author was consulted. Pain scores were reported within the range 0–10, either with the visual analgesic score (VAS) or numeric pain rating score (NRS). If pain scores in the included articles were reported on a different scale, they were converted to 0–10 for consistency. The primary descriptive statistic for pain scores was the mean (± standard deviation (sd)); if not reported, other descriptive statistics were noted. Postoperative complications are expressed as number with percentages (%). When available, significant differences between groups within studies were reported. If possible, missing data was obtained from the corresponding authors of the included manuscripts.

### Data analysis

All included studies were categorized according to the type of analgesia used: TEA, locoregional analgesia, and systemic analgesia. The feasibility of conducting a meta-analysis was evaluated by examining clinical (e.g. anaesthesiological and surgical procedures) and methodological (e.g. study design and risk of bias) differences across studies. Statistical heterogeneity was additionally assessed using the I^2^ statistic for the time points with the most frequently reported pain scores using the meta package in R (version 4.5.1). When I^2^ exceeded 75%, heterogeneity was considered substantial, and a comparative meta-analysis of TEA, locoregional analgesia and systemic analgesia was deemed inappropriate. In such cases, the results were synthesized for a narrative systematic review only.

## Results

### Quantity and quality of the studies

**Fig. 1:**
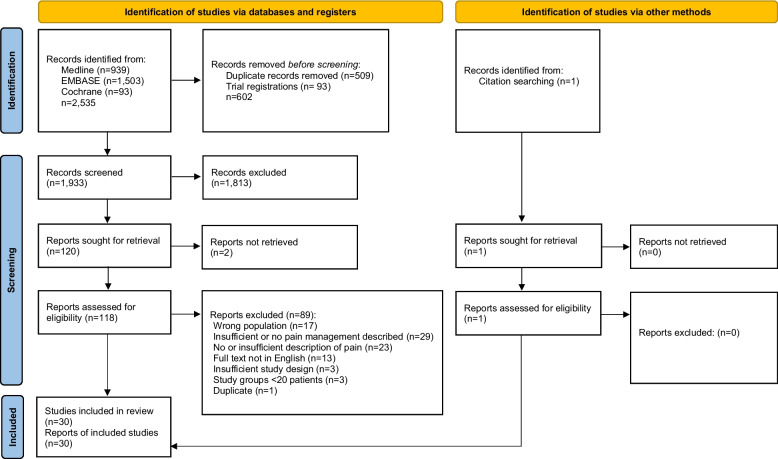
PRISMA 2020 flow diagram of study selection (*n* = number)

**Fig. 2:**
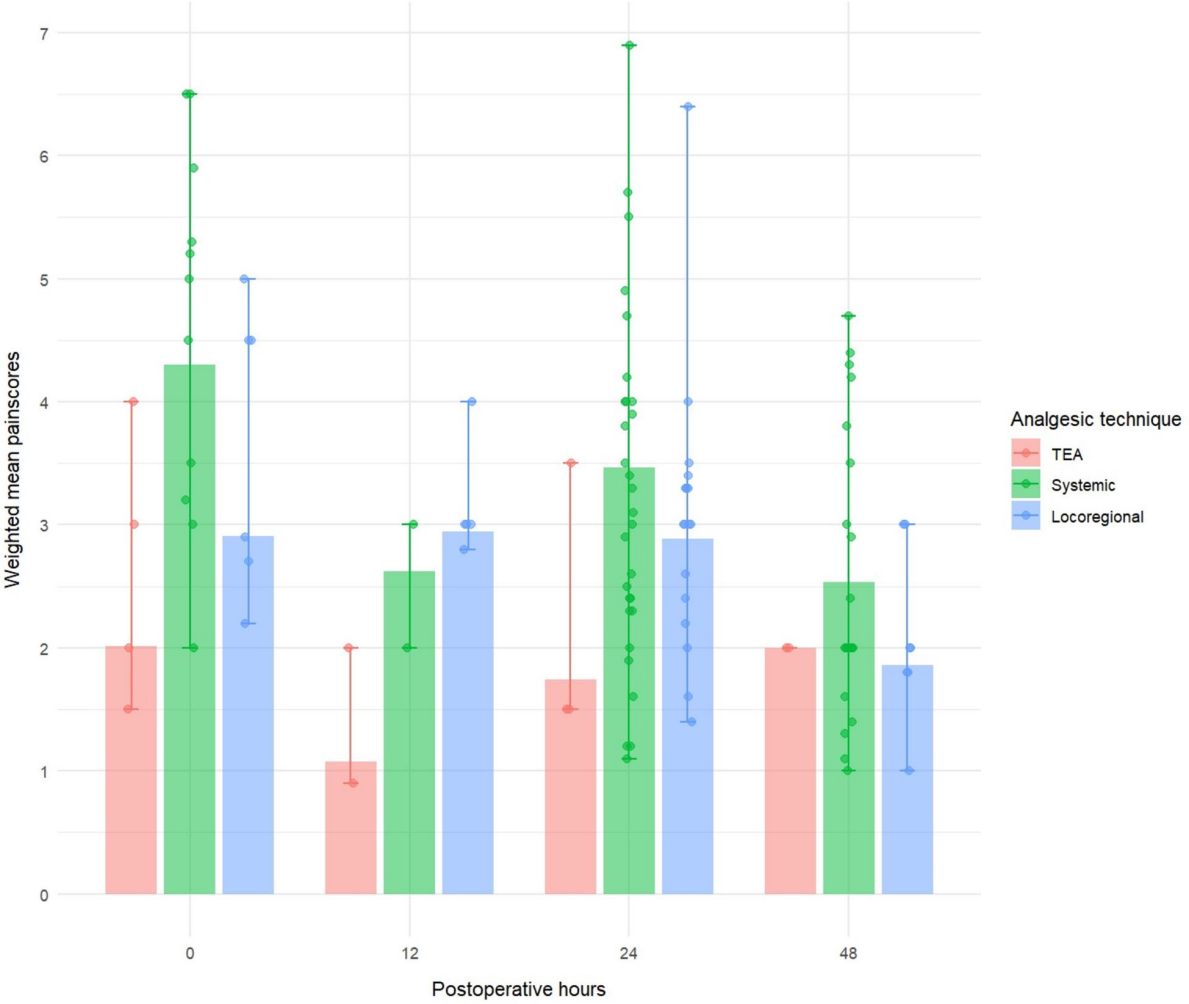
Illustration of weighted mean postoperative pain scores per analgesic technique Bar chart describing the weighted mean postoperative pain scores after VATS for spontaneous pneumothorax per time point at which pain was assessed. The most frequently reported time points were 0, 12, 24 and 48 h. The mean pain score with the range is depicted, weighted for the number of patients included in each article. The dots illustrate the individual study mean pain scores. The data shown in the figure were only from the studies who reported pain scores on the corresponding time points. TEA: thoracic epidural analgesia

A total of 2,535 records were identified through the database searches. After deduplication and removal of trial registrations, 1,933 records were screened of which 30 articles fulfilled the inclusion criteria, comprising 4 RCTs and 26 cohort studies (Fig. 1). Study characteristics and overall RoB are described in Table 1 and additional information is presented in supplemental file 4–5. In Table 2 an overview of the studies reporting analgesia-related postoperative complications is provided. The included studies comprised 3,203 patients undergoing VATS for SP. In the randomized studies (*n* = 4), 3 studies were scored as some concerns and one study as low RoB. The non-RCTs (*n* = 26) were scored as low RoB in 2 studies, moderate RoB in 21 studies and high RoB in 3 studies (Additional file 3 risk of bias assessment). Due to substantial differences in reporting of outcomes, surgical, anaesthesiological and analgesic strategies, both clinical and methodological heterogeneity were considered too high to justify a meta-analysis. Heterogeneity was additionally tested using I^2^ statistic for pain scores at 0, 12, 24 and 48 h, statistically confirming high heterogeneity (I^2^ > 96%). The level of heterogeneity was also calculated for the comparisons regional versus systemic and epidural versus locoregional analgesia, both of which likewise showed high heterogeneity (I^2^ > 84%). These findings precluded pooling of results for meta-analysis. Although no formal meta-analysis was performed, Fig. 2 demonstrates the weighted mean postoperative pain scores per analgesic approach at the most frequently reported time-points for illustration purposes.

**Table 1:** Study characteristics and overall RoB of included studies

Author, year and country	RCT*	N	Pleurodesis^a^	Block type	Block type characteristics	Anesthetic solution	Systemic analgesia details	Primary outcome NRS/VAS score^b^				RoB
								**0h**	**12h**	**24h**	**48h**	
Regional versus systemic analgesia												
Pompeo 2007 [17] Italy	Yes	43	Yes	TEA	Continuous	0.2–0.5% ropivacaine with 1ug/ml sufentanyl 2–5 ml/h	N/A	2.0 (± 3.0)	-	-	-	S
				Sys	-	-	Continuous infusion of tramadol and ketorolac	3.5 (± 2.0)^c^	-	-	-	
Liu 2022 [18] China	Yes	325	No	Loco	Intercostal incision local anesthesia, visceral pleural surface anesthesia and vagus nerve block	Not described	PCA morphine	-	-	2.4 (± 1.5)	1.8 (± 1.4)	L
				Sys	-	-	PCA morphine	-	-	2.4 (± 1.4)	1.6 (± 1.1)	
Xie 2020 [19] USA	No	66	Yes	TEA	Continuous	Bupivacaine or ropivacaine 0.1–0.2% bolus 3-15ml, then 0.15ml/kg/h with 2mcg/ml fentanyl or 10 mcg/ml hydromorphone	PCM. Rescue: NSAID, opioids or PCA	3.0 (±?)	2.0 (±?)	1.5 (±?)^c^	2.0 (±?)	M
				Loco	Ultrasound guided continuous PVB	Ropivacaine 0.2% 20–40 ml bolus, then 0.15ml/kg/h		2.7 (±?)	3.0 (±?)	3.3 (±?)	2.0 (±?)	
				Sys	-	-		2.0 (±?)	2.0 (±?)	3.3 (±?)	2.0 (±?)	
Allain 2019 [11] France	No	59	Yes	Sys	-	-	PCM, NSAID, opioids. Rescue: short acting opioid (when VAS > 3)	4.5 (2.0–7.0)^d^	3.0 (2.0–6.0) ^d^	3.0 (2.0–6.0)^d^	2.0 (1.5–4.0)^d^	M
				Loco	Continuous PVB	15 ml bolus lidocaine 20mg/ml and epinephrine 50 ug/ml, then ropivacaine 2mg/ml 10ml/h		5.0 (1.0–6.0)^d^	4.0 (2.0–5.0) ^d^	3.0 (2.0–4.0)^d^	2.0 (2.0–4.0)^d^	
				Loco	Ultrasound guided continuous SAPB	15 ml bolus lidocaine 20mg/ml and epinephrine 50 ug/ml, then ropivacaine 2mg/ml 10ml/h		4.5 (0.0–6.0)^d^	3.0 (2.0–5.5) ^d^	2.0 (1.5–4.5)^d^	2.0 (0.0–4.0)^d^	
				Loco	Ss-SAPB	20-30ml ropivacaine 2mg/ml		4.5 (0.5–7.0)^d^	3.0 (3.0–4.0) ^d^	3.0 (3.0–4.0)^d^	3.0 (2.0–4.0)^d^	
Li 2020 [20] China	No	68	Yes	Loco	ultrasound guided PVB	20 ml ropivacaine 0.5%	NSAID, opioids (VAS > 4)	-	-	1.4 (± 0.5)	-	M
				Sys	-	-		-	-	2.0 (± 1.1)	-	
Jung 2019 [21] Korea	No	104	No	Sys	-	-	N/A	2.35 (±?)	-	2.06 (±?)	1.91 (±?)	H
				Loco	Pre-emptive analgesia	1% lidocaine	opioids	2.15 (±?)	-	1.56 (±?)^c^	1.50 (±?)^c^	
Fernandez 2005 [22]	No	118	Yes	TEA	PCA	Fentanyl 5 ug/ml and bupivacaine 0.1%	N/A	-	-	0 [0–3]	-	H
UK				Loco	PCA occasionally with ICNB or PVB			-	-	0 [0–3]	-	
Epidural versus locoregional analgesia												
Ishikawa 2012 [23] Japan	Yes	40	No	TEA	-	0.375% ropivacaine 3 ml/h	NSAID	4.0 (±?)	-	3.5 (±?)	-	S
				Loco	Intrapleural analgesia	2 times bolus 40 ml 0.375% ropivacaine		2.9 (±?)	-	4.0 (±?)	-	
Spaans 2023 [10] The Netherlands	No	218	Yes	Loco	Multilevel ss-ICNB Th1-2 until Th7-9	15-20ml ropivacaine 2-10mg/ml or bupivacaine 5mg/ml	PCM, NSAID, opioids	2.2 (±?)	2.8 (±?)	3.0 (±?)	1.8 (±?)	M
				TEA	Continuous TEA	Ropivacaine 1-2mg/ml or bupivacaine 1.25–2.50 mg/ml with sufentanyl		1.5 (±?)	0.9 (±?)	1.5 (±?)	2.0 (±?)	
Studies evaluating single analgesic techniques												
Systemic analgesia												
Chen 2006 [24] Taiwan	No	202	Yes	Sys	-	-	PCM, NSAID, opioid (VAS > 7)	-	-	6.9(6.6–7.2)^c^	4.7 (4.4–5.0)	M
				Sys	-	-		-	-	5.5 (5.2–5.9)	4.4 (4.2–4.6)	
Freixinet 2004 [25] Spain	No	46	Yes	Sys	-	-	NSAID, opioids i.m. (VAS > 7)	-	-	3.8 (± 1.8)	2.9 (± 1.7)	M
Zhong 2024 [26] China	No	128	Yes	Sys	-	-	NSAID	-	-	4.0 (1.0)^d^	2.0 (1.0)^d^	M
			Yes	Sys	-	-		-	-	4.0 (1.0)^d^	2.0 (1.0)^d^	
Jeon 2016 [27] Korea	No	86	Yes	Sys	-	-	NSAID and PCA fentanyl	5.2 (± 1.5)	-	3.5 (± 1.6)	-	M
			Yes	Sys	-	-		6.5 (± 2.3)	-	4.2 (± 2.1)	-	
Yamaguchi 2021 [28] Japan	No	73	No	Sys	-	-	PCM, NSAID, PCA. Rescue: PCA and NSAID	5.3 (±?)	-	3.9 (±?)	3.5 (±?)	M
			No	Sys	-	-		5.9 (±?)	-	4.9 (±?)	4.2 (±?)	
Kawaguchi 2021 [29] Japan	No	42	No	Sys	-	-	24 h routine NSAID Rescue: PCM (i.v.), NSAID or opioids	6.5 (±?)	-	4.0 (±?)	-	M
			No	Sys	-	-		5.0 (±?)	-	1.9 (±?)^c^	-	
Horio 2002 [30] Japan	No	103	Yes	Sys	-	-	NSAID	-	-	1.2 (± 0.9)	-	M
			No	Sys	-	-		-	-	1.1 (± 0.9)	-	
Kagimoto 2024 [31] Japan	No	43	No	Sys	-	-	Routine NSAID. Rescue: PCM, NSAID i.v. and opioids	-	-	-	-	M
			No	Sys	-	-		-	-	-	-	
Rena 2008 [32] Italy	No	220	Yes	Sys	-	-	PCM and continuous opioids	3.0 (± 1.0)	-	2.4 (± 1.0)	2.0 (± 0.9)	M
			Yes	Sys	-	-		3.2 (± 0.9)	-	2.5 (± 1.0)	2.0 (± 0.9)	
Chen 2012 [33] Taiwan	No	160	Yes	Sys	-	-	PCM, nsaid. Rescue: opioids i.m. (VAS > 7)	-	-	4.7 [1.0–10.0]	3.8 [1.0–9.0]	M
			Yes	Sys	-	-		-	-	5.7 [3.0–10.0]^c^	4.3 [2.0–8.0]	
Wang 2016 [34] Taiwan	No	57	Yes	Sys	-	-	NSAID and opioids i.v. (VAS > 4)	-	-	1.2 (± 0.7)	1.3 (± 0.5)	L
			Yes	Sys	-	-		-	-	1.6 (± 1.0)	1.1 (± 0.9)	
			Yes	Sys	-	-		-	-	2.3 (± 0.8)^c^	1.4 (± 1.0)	
Masmoudi 2017 [35] France	No	351	Yes	Sys	-	-	PCM, nefopam and opioid. Rescue: PCA morphine	-	-	-	2.0 (± 1.7)	M
Hsu 2021 [36] Taiwan	No	204	Yes	Sys	-	-	PCM, NSAID, opioids every 4-6h (VAS > 7)	-	-	2.9 (± 2.0)	2.4 (± 1.8)	M
			Yes	Sys	-	-		-	-	2.6 (± 1.8)	2.0 (± 1.7)	
Kutluk 2018 [37] Turkey	No	135	Yes	Sys	-	-	PCM and NSAID	-	-	2.3 (± 0.2)	-	M
			Yes	Sys	-	-		-	-	3.1 (± 0.2)	-	
			Yes	Sys	-	-		-	-	3.4 (± 0.2)^c^	-	
Locoregional analgesia												
Kim 2021 [38] Korea	Yes	50	No	Loco	Ss-ICNB	5 ml 0.375% ropivacaine	PCM, NSAID (NRS 4–5) or opioid (NRS > 6)	-	-	3.4 (± 2.0)	-	S
				Loco	ultrasound guided SAPB	20 ml 0.375% ropivacaine		-	-	3.3 (± 1.7)	-	
Takamori 2024 [39] Japan	No	54	No	Loco	ICNB	Ropivacaine or levobupivacaine	N/A	-	-	3.0 (1.0–4.0)^d^	-	M
Kiriyama 2024 [40] Japan	No	74	No	Loco	ICNB	0.375% ropivacaine	PCM and NSAID	-	-	3.3 (±?)	-	M
			No	Loco	ICNB	0.375% ropivacaine		-	-	-	-	
Nachira 2018 [41] Italy	No	46	Yes	Loco	Multilevel ICNB Th2-8 under thoracoscopic visualization	Ropivacaine	N/A	-	-	3.5 (± 1.4)	-	M
			Yes	Loco	ICNB prior to incision and closure via the external chest wall	Ropivacaine		-	-	6.4 (± 2.5)^c^	-	
Hwang 2018 [42] Korea	No	41	No	Loco	pre-emptive analgesia and intrathoracic vagal block	20 ml 2% lidocaine	NSAID	-	-	2.6 (± 2.1)	-	L
			No	Loco	pre-emptive analgesia and intrathoracic vagal block	20 ml 2% lidocaine		-	-	1.6 (± 1.3)	-	
Tsuboshima 2016 [43] Japan	No	25	No	Loco	Continuous PVB	Bolus 20 ml levobupivacaine 0.25%, then 4ml/h 0.17%	PCA if necessary	-	-	2.2 (± 1.6)	1.0 (± 1.0)	M
Hyland 2001 [44] Canada	No	22	Yes	Loco	(continuous) ICNB	Bupivacaine hydrochloride	PCA if necessary	3 (±?)	-	-	-	H

^*^RCTs regarding analgesic techniques

^a^if pleurodesis is not performed, the procedure is limited to bullectomy

^b^presented in mean ± sd or mean [range] or mean (95%-CI) or numbers with

^d^ are presented in median (low IQR – high IQR) or median (IQR)

^c^ significant difference between groups within studies. *N* number of included patients, *Loco* locoregional, *Sys* systemic, *TEA* thoracic epidural analgesia, *(ss)-ICNB* (single-shot) intercostal nerve block, *SAPB* serratus anterior plane block, *PCA* patient controlled analgesia, *PCM* paracetamol or acetaminophen, *NSAID* non-steroidal anti-inflammatory drugs, *Th* thoracic level, *RoB* risk of bias, *h* hour, *NRS* numeric pain rating score, *VAS* visual analgesic score, *POD* postoperative day, *SD* standard deviation, *N/A* not described, *L* low risk of bias, *S* some concerns, *M* moderate risk of bias, *H* high risk of bias. If doses of analgesia are not reported, it is not presented by the article

**Table 2:** Reported analgesia related complications in the included studies

Author	Type of analgesia	Morphine consumption	Postoperative complications
Regional versus systemic analgesia			
Pompeo 2007 [17]^b^	1. TEA 2. Systemic analgesia	-	1. Vomiting 1 (4.8%), urinary retention 1 (4.8%), no mortality or major morbidity 2. No vomiting, urinary retention, mortality or major morbidity
Liu 2022 (18)^a^	1. Intercostal incision local anesthesia, visceral pleural surface anesthesia and vagus nerve block 2. Systemic analgesia	PCA morphine 1mg/ml. No difference in morphine use	1. Nausea 3.70%, Urinary retention 0.62% 2. Nausea 3.07%, Urinary retention 0%
Xie 2020 [19]	1. TEA 2. Ultrasound guided PVB 3. Systemic analgesia	Opioid use lower in TEA group on POD0-1. No difference in PCA use	No severe advents related to TEA or PVB No difference in nausea TEA significantly more often use of Nalbuphine for itching on POD0-1
Allain 2019 [11]^b^	1. Systemic analgesia 2. Continuous PVB 3. Ultrasound guided continuous SAPB 4. Ss-SAPB	No difference in overall morphine consumption, but in group 1 more high-dose morphine (> 50 mg) during first 72 h	1. PONV 3 (21%), urinary retention 1 (7%) 2. PONV 2 (22%), urinary retention 2 (22%) 3. PONV 8 (42%), urinary retention 0 (0%) 4. PONV 2 (13%), urinary retention 1 (6%)
Epidural versus locoregional analgesia			
Ishikawa 2012 [23]^a^	1. TEA 2. Intrapleural analgesia	-	1. Headache 1 (5%), nausea 3 (15%), no urinary retention, hypoxia and hypotension 2. Headache 1 (5%), nausea 2 (10%), no urinary retention, hypoxia and hypotension
Spaans 2023 [10]^a^	1. multilevel ss-ICNB 2. TEA	No difference in mean opioid dose, but the proportion of patients in group 1 requiring opioids was higher on POD0-1	1. No urinary infection, cardiac arrhythmia, or other infection 2. Urinary infection 1 (1%), cardiac arrhythmia 1 (1%), other infection 3 (3%)
One analgesic technique assessed			
Kim 2021 [38]^b^	1. ss-ICNB 2. ultrasound guided SAPB	-	1. dizziness 2 (8%), PONV 1 (4%) 2. dizziness 2 (8%), PONV 1 (4%)
Hwang 2018 [42]^a^	1. Pre-emptive analgesia and intrathoracic vagal block with intubation 2. Pre-emptive analgesia and intrathoracic vagal block with intubation without intubation	-	1. Headache 1 h 1 (5%); 24 h 1 (5%) Nausea 1 h 2 (10%); 24 h 1 (5%). No vomiting, bradycardia or hypotension 2. Headache 1 h 4 (19%); 24 h 2 (9.5%). Nausea 1 h 1 (4.8%); 24 h 1 (4.8%). Bradycardia 1 h 1 (4.8%); 24 h 1 (4.8%). No vomiting, bradycardia or hypotension
Tsuboshima 2016 [43]	Continuous PVB		No urinary retention or hypotension

The exact number of each complication is reported with its respective percentage, using all included patients in the denominator

*TEA* thoracic epidural analgesia, *PVB* paravertebral block, *(ss)-SAPB* (single shot) serratus anterior plane block, *PONV* postoperative nausea and vomiting, *ss-ICNB* single shot intercostal nerve block, *POD* postoperative day

^a^No significant differences were seen within the study

^b^No statistical test was performed within the study

### Regional versus systemic analgesia

Two RCTs and five observational studies compared regional analgesia with systemic analgesia [11, 17–20]. The RCT from Pompeo et al. was assessed as having some concerns. Continuous TEA (*n* = 21) was compared against continuous systemic infusion of tramadol and ketorolac (*n* = 22) after multiportal VATS bullectomy with abrasion [17]. Patients assigned for TEA had awake surgery, whereas patients with systemic analgesia received general anesthesia. Primary outcomes were technical feasibility and patient satisfaction. Pain scores at the end of surgery were lower in the TEA group (VAS 2 versus 3.5). No major complications were reported; minor complications in the TEA group included vomiting and urinary retention (4.8%). In the RCT by Liu et al., patients undergoing uniportal VATS bullectomy were assigned to perioperative spontaneous ventilation with locoregional analgesia (intercostal block at the incision site, visceral pleural anesthesia, and vagus nerve blockade) (*n* = 162) or mechanical ventilation without additional regional techniques (*n* = 163) and was assessed as low RoB [18]. Both groups received postoperative patient-controlled analgesia (PCA) with morphine. The primary outcomes were safety and feasibility. Pain scores over the first 24 h (VAS 0–10) were comparable (3.16 to 2.39 vs. 3.32 to 2.36), as well as morphine consumption and complications.

The following three comparative observational studies were all assessed as moderate RoB. In a single-center retrospective case series, Xie et al. evaluated TEA (*n* = 23), ultrasound-guided continuous PVB (*n* = 34) and systemic analgesia (*n* = 9) after VATS pleurodesis [19]. All patients received standardized multimodal analgesia. Opioid consumption was lower after TEA. Pain scores (NRS 0–10) on POD1 were significantly lower in the TEA group (NRS 1.5) compared to PVB (NRS 3.3) and systemic analgesia (3.4). Patients in the TEA group needed nalbuphine for pruritus more often. No severe adverse events were reported.

Allain et al. compared systemic analgesia (*n* = 15), continuous PVB (*n* = 9), ultrasound guided continuous SAPB (*n* = 19) and ss-SAPB (*n* = 16) after uniportal VATS bullectomy and chemical pleurodesis in a single-center prospective setting [11]. All patients received standardized multimodal analgesia and rescue medication consisted of oral morphine. Total morphine consumption was similar; however, high-dose morphine use (> 50 mg within 72 h) occurred more frequently in the systemic analgesia group (*p* = 0.02) and low-dose use (< 50 mg) more in the continuous SAPB group (*p* = 0.06). Pain scores (VAS 0–10) decreased over 24 h in all groups (systemic: 4.5 to 3; PVB: 5 to 3; continuous SAPB: 4.5 to 2; ss-SAPB: 4.5 to 3), without significant differences. No differences in PONV and urinary retention were found.

Li et al. conducted a retrospective study in patients undergoing 2port VATS bullectomy with chemical pleurodesis, comparing tracheal intubation with controlled ventilation and systemic analgesia (n = 34) versus laryngeal mask spontaneous ventilation with ultrasound-guided PVB (n = 34) [20]. All patients received NSAIDs, and subcutaneous morphine as rescue medication. Pain scores at 3 h postoperatively were lower in the PVB group (0.9 vs. 2.0, p = 0.024), but no difference was observed at 24 h.

Two observational studies were assessed as having high RoB. The first compared pre-emptive analgesia (n = 52) with systemic analgesia alone (n = 52) and found significantly lower pain scores on POD1 and 2 in the pre-emptive group [21]. The second study compared TEA (n = 22) with PCA, occasionally combined with ICNB or PVB (n = 96) [22]. While pain scores on POD1 were comparable, the time to transition to oral analgesia alone was significantly longer in the TEA group (p = 0.001). Both studies were judged to be at high RoB in the domain “classification of interventions”, as the anesthetic regimen was insufficiently described or additional techniques were used without adequate clarification.

Although in general it appears that patients receiving regional analgesia have lower pain scores and a reduced morphine consumption compared to systemic analgesia alone, the certainty of evidence was considered very low, due to lack of high-quality RCTs, presence of heterogeneity, indirectness and risk of bias.

### Epidural versus locoregional analgesia

One RCT and two observational studies compared TEA with locoregional analgesia; all were assessed as having some concerns or moderate RoB [10, 19, 23]. In the RCT by Ishikawa et al., continuous TEA (n = 20) was compared with intrapleural analgesia (n = 20) using ropivacaine boluses through the chest tube after multiportal VATS bullectomy [23]. Diclofenac served as postoperative rescue medication. The postoperative pain scores were measured 6 times a day by VAS. The mean pain score was ≤ 4.0 in the first 24 h in both groups, without significant differences. Analgesia-related complications were comparable (Table 2). A multicenter retrospective study compared ss-ICNB (n = 96) versus TEA (n = 122) after VATS bullectomy and pleurectomy [10]. Patients received postoperative multimodal analgesia. The mean opioid dose was similar; on POD0-1, a higher proportion of patients in the ss-ICNB group required opioids. The percentage of pain scores with NRS ≥ 4 from POD0-3 was similar between ss-ICNB and TEA (11.1% versus 14.3%; p = 0.24). No differences in postoperative complications were observed. Lastly, in the aforementioned retrospective study by Xie et al. patients receiving TEA had significantly lower pain scores and opioid consumption compared to PVB [19].

Although TEA and locoregional analgesia appear equally effective in general, the certainty of evidence was considered very low, due to lack of high-quality RCTs, presence of heterogeneity, indirectness and risk of bias.

### Studies evaluating single analgesic techniques

The remaining 21 studies only described one type of analgesic technique: either systemic or locoregional. One was a RCT comparing two different locoregional techniques [38], whereas the remainder were observational and not designed to compare analgesic techniques.

#### Systemic analgesia only

In 14 studies [24–37], postoperative pain after VATS for SP was managed using only systemic analgesia. The majority of all reported mean or median pain scores was lower than 4, with the highest pain scores measured during the first postoperative day (varying from 1–7 among studies). From 24 h postoperatively onwards, mean pain scores were generally below 3 (66% of studies). Eleven studies used a multimodal analgesic regime consisting of acetaminophen and/or NSAIDs, and opioids in case of insufficient pain control [24, 25, 27–29, 31–36] and three studies used NSAIDs without opioids as rescue medication [26, 30, 37].

Most studies employing systemic analgesia were small and designed to retrospectively compare uniportal and multiportal VATS, or to evaluate different pleurodesis techniques. The results suggested lower pain scores in case of subxiphoidal or uniportal surgical approach, and in case of mechanical instead of chemical pleurodesis [24, 26, 30, 32–35, 37]. None of the studies reported postoperative complications regarding systemic analgesia.

#### Locoregional analgesia only

The RCT by Kim et al. compared ss-ICNB (n = 25) with ultrasound guided SAPB (n = 25) after uniportal VATS bullectomy and was assessed as having some concerns regarding RoB [38]. All patients received postoperative multimodal analgesia. Patient with ss-ICNB received significantly more ketorolac compared to SAPB. The mean NRS (0–10) scores decreased in the ss-ICNB from 3.9 to 3.4 within the first 12 postoperative hours and in the SAPB group from 4.0 to 3.3, without significant differences.

The remaining six observational studies evaluated single locoregional techniques. Four studies employed an ICNB (all lacking technical details), one study an intrathoracic vagal block and one study a PVB [39–43]. Pain scores in the first postoperative hours varied between 2–6 and dropped to 1–3 after 24 h onwards. Continuous PVB showed similar pain outcomes compared with single-shot techniques; 4% of patients additionally received patient-controlled analgesia with opioids [43]. Postoperative complications were only reported by Tsuboshima et al., specifically noting the absence of urinary retention and hypotension [43]. The study by Hyland et al. evaluated the use of ICNB and reported a mean pain score of 3 immediately after surgery [44]. This study was assessed as having a high RoB in the domain “measurement of outcomes” as patients were asked, up to 6 years postoperatively, to retrospectively report their pain score immediately after surgery.

## Discussion

This systematic review evaluated the effectiveness of various analgesic strategies for managing pain following VATS for SP. We found considerable clinical heterogeneity among the included studies with respect to surgical and anesthetic details, as well as the technical characteristics of locoregional or systemic analgesia. Methodological and statistical heterogeneity were also substantial, with only four small RCTs, most of which had some concerns regarding RoB, and the vast majority of studies not being designed to compare analgesic techniques. Given these limitations, a quantitative meta-analysis was not appropriate, compelling only a narrative assessment of individual studies. Nevertheless, despite the paucity of high-quality studies, this systematic review provides a comprehensive overview of the existing literature and aims to establish an evidence-based foundation for future research in this field. Our findings suggest that both locoregional analgesia and TEA are associated with favorable pain scores compared to systemic analgesia alone. Pain scores are highest during the first postoperative day, whereas by POD2 the measured pain scores typically drop below 3 across all types of analgesia.

Although TEA is the historic standard after surgery for pneumothorax, only 4 of our included studies have evaluated TEA: two RCT’s and two observational studies [10, 17, 19, 23]. The RCT by Ishikawa compared TEA with intermittent intrapleural application of ropivacaine and reported no differences in pain scores [23]. However, intermittent intrapleural anesthesia is an uncommon and labor-intensive local technique, and may increase infections due to frequent disconnection of the chest tube, impeding widespread implementation. Another RCT by Pompeo found that awake surgery with TEA provided better pain relief than systemic analgesia during general anesthesia [17]. By including differences in general anesthetic approach in the study design, the reliability of comparing analgesic techniques was reduced. In addition to the limitations of the RCTs, the non-randomized comparative studies provided conflicting results regarding the benefits of TEA. Although TEA was associated with slightly lower pain scores and reduced opioid consumption compared to PVB and systemic analgesia in one retrospective series [19], another retrospective study demonstrated comparable pain scores and opioid dose between TEA and ss-ICNB [10]. Despite lack of high-level of evidence and inconsistent findings, the results suggest that both TEA and ICNB are acceptable techniques for pain management in SP patients.

However, the PROSPECT guideline recommends PVB or ESPB over TEA, with SAPB as optional alternative, while ICNB and intrapleural analgesia are not even mentioned. This recommendation is based on a Delphi consensus assuming sufficient analgesic efficacy of locoregional analgesia without the TEA-related side effects [7]. The studies including TEA in our review, however, reported no significant increase in complications, except for the occurrence of pruritus [19]. Moreover, it is important to note that the PROSPECT guideline is not specifically tailored to SP patients. Despite locoregional techniques appearing to be effective following surgery for SP, selecting the optimal technique is challenging, given the wide range of available techniques and the current lack of high-quality comparative studies. Although the RCT by Liu reported comparable pain scores at 24 and 48 h between ICNB and systemic analgesia, the mode of ventilation was different among randomized groups, precluding conclusions on analgesic techniques [18]. The single prospective comparative study reported comparable pain scores after continuous PVB, continuous SAPB, ss-SAPB or systemic analgesia, but significantly higher morphine consumption in patients receiving only systemic analgesia [11]. Which locoregional technique to prefer therefore remains a knowledge gap.

Interestingly, the majority of the included studies in this systematic review utilized only systemic analgesia following VATS for SP. Although many studies reported acceptable pain scores during the first postoperative days, five studies documented relatively high pain scores, indicating inadequate pain control [24, 27, 28, 33, 37]. This contrasts with studies in which locoregional or epidural techniques were used alongside systemic analgesia, demonstrating lower postoperative pain scores during the initial postoperative period. Notably, the primary focus of these studies was not the evaluation of systemic analgesia itself, but other perioperative factors such as the number of surgical ports, the addition of chemical pleurodesis or postoperative rehabilitation. The heterogeneity in study design and endpoints limits the ability to draw firm conclusions regarding the effectiveness of systemic analgesia only.

Nevertheless, reliance on systemic analgesia alone stands in contrast with guidelines for postoperative pain management, which recommend the use of multimodal analgesia, defined as the combined use of various analgesic medications and techniques [7, 45]. In general, cumulative opioid consumption is higher when systemic analgesia alone is used, as also demonstrated in this review, which may increase the risk of opioid related side-effects such as constipation, dependence and PONV. Interestingly, despite the higher morphine consumption in this group, no differences in PONV were observed across RCTs and observational studies; however, event numbers were limited, precluding meaningful comparisons. Nevertheless, PONV may negatively impact recovery and patient satisfaction. Multimodal analgesia is therefore recommended to optimize pain control through additive effects of different types of analgesics, while minimizing the side effects of individual drugs [43]. This approach may reduce opioid consumption and its side effects, thereby supporting ERATS goals such as PONV prevention, early mobilization and early oral diet [6, 46]. Although few studies reported on analgesia-related side effects, no significant differences in PONV between the various analgesic techniques were observed [10, 11, 17–19, 23, 38, 42, 43]. In general, a multimodal analgesic regimen consists of acetaminophen, NSAIDs, and opioids, alongside a locoregional or epidural technique [7, 45]. However, some studies reported no systemic multimodal regime at all [17, 39, 41] or described the use of NSAIDs or opioids only [18, 23, 26, 42, 43], thereby overlooking the added value of a multimodal approach.

When selecting the optimal analgesic technique, factors beyond pain scores and opioid consumption should be considered, including surgical and patient-related characteristics. This review included two studies evaluating the combination of chemical pleurodesis and mechanical abrasion, which reported significantly higher pain scores compared to studies employing a single pleurodesis method or bullectomy only [24, 33]. Additionally, several studies, including one study rated as low RoB [34], associated multiport VATS with higher pain scores compared to uniportal VATS [34, 37, 41]. Patient-related factors, such as comorbidities and social determinants, are likewise crucial for tailoring effective postoperative pain management [45]. We did not perform a subgroup analysis of all these possible confounding factors, as it was not our primary objective and our search strategy was not designed to capture such data. All these factors should however be carefully considered and, due to the lack of high level of evidence, shared decision-making is likely the most appropriate approach for selecting the optimal analgesic strategy at this moment.

Although this is the first review to specifically provide an overview of analgesic techniques after VATS for SP, some limitations need to be addressed. Only a minority of the included studies were comparative, and even fewer were randomized. Furthermore, most studies were assessed as having moderate or some concerns regarding risk of bias and there was substantial heterogeneity between studies in terms of study design, surgical procedures and analgesic regimens. As a result, performing a meta-analysis was not appropriate. Lastly, postoperative pain scores were selected as the primary outcome measure in this review, as they are commonly used in clinical practice to monitor pain. However, pain scores represent only a single dimension of recovery. As previously discussed, analgesic techniques impact a range of other outcomes beyond pain, including mobilization and PONV, which also significantly affect patient recovery. These factors are crucial in determining the optimal analgesic strategy and should therefore be considered. Future studies may benefit from incorporating alternative outcome measures that assess multiple domains, such as pain, comfort, and independence, like the Quality of Recovery-15 (QoR-15) questionnaire, to more comprehensively evaluate the effectiveness of analgesic techniques after surgery [47].

## Conclusions

The overall certainty of evidence supporting TEA or locoregional analgesia over systemic analgesia alone, as well as of TEA over locoregional analgesia, was rated as very low due to bias, inconsistency (heterogeneity), indirectness, and imprecision. Both surgical and patient related factors should be taken into consideration when counselling the patient on analgesia, with an important role for shared decision-making. In addition, the current evidence suggests the use of a multimodal analgesic approach, including acetaminophen and short-term opioids, combined with either locoregional or epidural analgesia following surgery for SP. Lastly, these findings lay the groundwork for future high-quality research that should ideally incorporate multiple domains to comprehensively determine the most effective analgesic technique, and facilitate the development of evidence-based recommendations.

## Supplementary Information

Additional file 1. PRISMA 2020 checklist, Description of the data: the completed PRISMA 2020 checklist is shown.

Additional file 2. Search strategy, Description of the data: the complete search strategy is presented.

Additional file 3. Risk of bias assessment per study, Description of the data: the complete risk of bias assessment is presented regarding the primary outcome measure pain scores.

Additional file 4. All reported postoperative pain scores until 48 hours after surgery per included artic.

Additional file 5. Surgical procedure, analgesic technique and multimodal medications per included article.

## Data Availability

All data generated or analysed during this study are included in this published article and its supplementary information files.

## Abbreviations

ERATS: Enhanced recovery after thoracic surgery
ESPB: Erector spinae plane block
ICNB: Intercostal nerve block
LOS: Length of hospital stay
NRS: Numeric pain rating score
NSAID: Non-steroidal anti-inflammatory drugs
PCA: Patient-controlled analgesia
POD: Postoperative day
PONV: Postoperative nausea and vomiting
PVB: Paravertebral block
RCT: Randomized controlled trial
RoB: Risk of bias
SAPB: Serratus anterior plane block
SD: Standard deviation
SP: Spontaneous pneumothorax
Ss: Single-shot
TEA: Thoracic epidural analgesia
VAS: Visual analgesic score
VATS: Video-assisted thoracoscopic surgery
