# Supplementary material for: Postoperative pain management after VATS for spontaneous pneumothorax - a systematic review
Source: BMC Anesthesiol. 2026 May 5;26:377. doi: 10.1186/s12871-026-03865-1 (PMC13289495; doi:10.1186/s12871-026-03865-1)
Supplement: Supplementary file 2 — Additional file 2. Search strategy, Description of the data: the complete search strategy is presented. [file 12871_2026_3865_MOESM2_ESM.docx]

# **Additional file 2. Search strategy**

**MEDLINE (Ovid):**

| **ID** | **Search** | **Hits** |
| --- | --- | --- |
| **1** | exp pneumothorax/ or (pneumothora* or (lung* adj3 collaps*)).ab,ti,kw. | 35,295 |
| **2** | exp pleurodesis/ or (pleurectom* or (pleura* adj3 (excision* or resection*)) or pleurodes* or bullectom* or abrasi* or talca* or (surger* adj3 pneumothora*)).ab,ti,kw. | 21,885 |
| **3** | exp pain/ or exp analgesia/ or exp postoperative period/ or exp pain management/ or exp postoperative care/ or exp pain measurement/ or exp nerve block/ or exp drug administration routes/ or exp drug therapy/ or (pain* or ((chest* or thora*) adj3 discomfort*) or analges* or ((post operat* or postoperat* or self) adj3 an*esthes*) or antinocicept* or ((postoperat* or post operat*) adj3 (period* or phase* or care* or therap* or treatment*)) or ((post an*esthes* or postan*esthes*) adj3 care) or visual analog scale* or nerve block* or autonomic block* or (drug adj3 (administrat* or infiltrat* or inject* or readministrat* or analys* or characterizat* or composit* or profil* or separate* or comparis* or therap*)) or (pharmac* adj3 (analys* or therap* or treat*)) or (medic* adj3 (therap* or treat*)) or medicat* or pharmacotherap* or pharmacotreat* or (therapeutic* adj3 use*)).ti,ab,kw. | 3,665,732 |
| **4** | 1 AND 2 AND 3 NOT ((exp animal/ or nonhuman/) NOT exp human/) | 1,045 |
| **5** | limit 4 to yr="1995 -Current" | 939 |

**EMBASE.com:**

| **ID** | **Search** | **Hits** |
| --- | --- | --- |
| #1 | 'pneumothorax'/exp OR 'primary spontaneous pneumothorax'/exp OR 'secondary spontaneous pneumothorax'/exp OR 'pneumothora*':ti,ab,kw OR ((lung* NEAR/3 collaps*):ti,ab,kw) | 72,683 |
| #2 | 'pleurectomy'/exp OR 'pleurodesis'/exp OR 'bullectomy'/exp OR 'abrasion'/exp OR ('pleurectom*' OR (pleura* NEAR/3 (excision* OR resection*)) OR 'pleurodes*' OR 'bullectom*' OR 'abrasi*' OR 'talca*' OR 'talka*' OR (surger* NEAR/3 pneumothora*) ):ti,ab,kw | 35,884 |
| #3 | 'pain'/exp OR 'analgesia'/exp OR 'postoperative period'/exp OR 'postanesthesia care'/exp OR 'postoperative care'/exp OR 'pain measurement'/exp OR 'analgesic agent'/exp OR 'pain control'/exp OR 'pain reduction'/exp OR 'pain assessment'/exp OR 'perioperative pain'/exp OR 'nerve block'/exp OR 'drug administration'/exp OR 'drug analysis'/exp OR 'drug comparison'/exp OR 'drug therapy'/exp OR 'pain*':ti,ab,kw OR (((chest* OR thora*) NEAR/3 discomfort*):ti,ab,kw) OR 'analges*':ti,ab,kw OR ((('post operat*' OR postoperat* OR self) NEAR/3 an*esthes*):ti,ab,kw) OR 'antinocicept*':ti,ab,kw OR (((postoperat* OR 'post operat*') NEAR/3 (period* OR phase* OR care* OR therap* OR treatment*)):ti,ab,kw) OR ((('post an*esthes*' OR postan*esthes*) NEAR/3 care):ti,ab,kw) OR 'visual analog scale*':ti,ab,kw OR 'nerve block*':ti,ab,kw OR 'autonomic block*':ti,ab,kw OR ((drug NEAR/3 (administrat* OR infiltrat* OR inject* OR readministrat* OR analys* OR characterizat* OR composit* OR profil* OR separate* OR comparis* OR therap*)):ti,ab,kw) OR ((pharmac* NEAR/3 (analys* OR therap* OR treat*)):ti,ab,kw) OR ((medic* NEAR/3 (therap* OR treat*)):ti,ab,kw) OR 'medicat*':ti,ab,kw OR 'pharmacotherap*':ti,ab,kw OR 'pharmacotreat*':ti,ab,kw OR ((therapeutic* NEAR/3 use*):ti,ab,kw) | 9,428,541 |
| #4 | #1 AND #2 AND #3 NOT ‘conference abstract’/it NOT ([animals]/lim NOT [humans]/lim) AND [1995-2025]/py | 1,503 |

**Cochrane Library:**

| **ID** | **Search** | **Hits** |
| --- | --- | --- |
| #1 | (pneumothora* OR (lung NEXT collaps*)):ti,ab,kw | 3,346 |
| #2 | (pleurectom* OR pleurodes* OR bullectom* OR abrasi* OR talca* OR  (pleura* NEXT (excision* OR resection*)) OR ('surgery for’ NEXT pneumothorax*)):ti,ab,kw | 2,296 |
| #3 | (pain* OR analges* OR antinocicept* OR pharmacotherap* OR pharmacotreat* OR (self NEXT anesthes*) OR ((chest OR thoracic OR thorax) NEXT (discomfort* OR pain*)) OR ((‘post operative’ OR postoperative) NEXT (anesthes* OR period* OR phase* OR care* OR therap* OR treatment*)) OR ((‘post anesthesia’ OR postanesthesia) NEXT care*) OR (‘visual analog’ NEXT scale*) OR ((nerve OR autonomic) NEXT block*) OR (drug NEXT (administrat* OR infiltrat* OR inject* OR readministrat* OR analys* OR characterizat* OR composit* OR profil* OR separate* OR comparis* OR therap*)) OR ((medicament OR pharmaceutical OR pharmacological) NEXT (therap* OR treat*)) OR (therapeutic NEXT use*) OR ((pharmaceutical OR pharmacological) NEXT analys*)):ti,ab,kw | 857,068 |
| #4 | #1 AND #2 AND #3 | 93 |
