# Supplementary material for: Postoperative pain management after VATS for spontaneous pneumothorax - a systematic review
Source: BMC Anesthesiol. 2026 May 5;26:377. doi: 10.1186/s12871-026-03865-1 (PMC13289495; doi:10.1186/s12871-026-03865-1)
Supplement: Supplementary file 3 — Additional file 3. Risk of bias assessment per study, Description of the data: the complete risk of bias assessment is presented regarding the primary outcome measure pain scores. [file 12871_2026_3865_MOESM3_ESM.docx]

# **Additional file 3. Risk of bias assessment per study**

| Study | Risk of bias tool used | Randomization process | Confounding | Selection of participants | Classification of interventions | Deviations from intended interventions | Missing outcome data | Measurement of the outcome | Selection of the reported result | Overall |
| --- | --- | --- | --- | --- | --- | --- | --- | --- | --- | --- |
| *Studies regarding pain management (N=30 studies)* | | | | | | | | | | |
| Pompeo (2007) | RoB 2 | S |  |  |  | S | S | S | S | S |
| Liu (2022) | RoB 2 | L |  |  |  | L | L | L | L | L |
| Ishikawa (2012) | RoB 2 | S |  |  |  | S | S | S | S | S |
| Kim (2021) | RoB 2 | L |  |  |  | S | S | L | S | S |
| Xie (2020) | ROBINS-I |  | M | M | L | L | NI | L | L | M |
| Allain (2019) | ROBINS-I |  | M | L | L | L | NI | L | L | M |
| Li (2020) | ROBINS-I |  | M | L | L | L | NI | L | L | M |
| Spaans (2023) | ROBINS-I |  | M | L | M | L | NI | L | L | M |
| Chen (2006) | ROBINS-I |  | NA | M | L | L | NI | M | L | M |
| Freixinet (2004) | ROBINS-I |  | NA | L | M | L | NI | L | M | M |
| Fernandez (2005) | ROBINS-I |  | M | M | H | NI | NI | M | M | H |
| Zhong (2024) | ROBINS-I |  | NA | M | M | L | NI | M | L | M |
| Jung (2019) | ROBINS-I |  | L | M | H | L | NI | M | M | H |
| Jeon (2016) | ROBINS-I |  | NA | M | M | L | NI | L | M | M |
| Yamaguchi (2021) | ROBINS-I |  | NA | M | M | L | NI | L | L | M |
| Kawaguchi (2021) | ROBINS-I |  | NA | L | M | L | NI | L | L | M |
| Horio (2002) | ROBINS-I |  | NA | L | L | L | NI | L | M | M |
| Kagimoto (2024) | ROBINS-I |  | NA | M | M | L | NI | L | L | M |
| Rena (2008) | ROBINS-I |  | NA | L | L | L | NI | M | L | M |
| Chen (2012) | ROBINS-I |  | NA | L | L | L | NI | M | L | M |
| Wang (2016) | ROBINS-I |  | NA | L | L | L | NI | L | L | L |
| Masmoudi (2017) | ROBINS-I |  | NA | L | L | M | NI | L | L | M |
| Hsu (2021) | ROBINS-I |  | NA | L | M | L | NI | M | L | M |
| Kutluk (2018) | ROBINS-I |  | NA | L | M | L | NI | L | L | M |
| Kiriyama (2024) | ROBINS-I |  | NA | L | M | L | NI | L | M | M |
| Takamori (2024) | ROBINS-I |  | NA | L | M | NI | L | M | L | M |
| Hyland (2001) | ROBINS-I |  | NA | L | M | NI | L | H | L | H |
| Hwang (2018) | ROBINS-I |  | NA | L | L | L | NI | L | L | L |
| Tsuboshima (2016) | ROBINS-I |  | NA | L | L | L | NI | M | M | M |
| Nachira (2018) | ROBINS-I |  | M | L | M | NI | NI | M | M | M |

Risk of Bias assessment regarding the primary outcome measure pain scores. The ROBINS-1 tool was used to evaluate the cohort studies. Studies with no direct comparison between analgesic techniques were assessed as single-arm cohort studies irrespective of the initial study design. Overall low risk of bias was defined as no items with moderate or high risk of bias. L = low risk of bias, S = some concerns, M = moderate risk of bias, H = high risk of bias, NI = no information an NA = not applicable.
