# Supplementary material for: Postoperative pain management after VATS for spontaneous pneumothorax - a systematic review
Source: BMC Anesthesiol. 2026 May 5;26:377. doi: 10.1186/s12871-026-03865-1 (PMC13289495; doi:10.1186/s12871-026-03865-1)
Supplement: Supplementary file 4 — Additional file 4. All reported postoperative pain scores until 48 hours after surgery per included artic. [file 12871_2026_3865_MOESM4_ESM.docx]

# **Additional file 4. All reported postoperative pain scores until 48 hours after surgery per included article**

| Study | Block | Pain measurements until 48 postoperative hours | |
| --- | --- | --- | --- |
| **Regional versus systemic analgesia** | | | |
| Pompeo 2007 (17) | 1. TEA  2. Sys | *Mean VAS score (range unknown) at end of surgery*  1. 2.0 (±3.0)  2. 3.5 (±2.0)^c^ | |
| Liu 2022 (18) | 1. Loco  2. Sys | *Mean VAS score (range unknown)*  1. 4h 3.2 (±1.7), 24h 2.4 (±1.5), 48h 1.8 (±1.4)  2. 4h 3.3 (±1.8), 24h 2.4 (±1.4), 48h 1.6 (±1.1) | |
| Xie 2020 (19) | 1. TEA  2. Loco  3. Sys | *Mean NRS score 0-10*  1. POD0 day 3.0 (±?), night 2.0 (±?), POD1 day 1.5 (±?)^c^, night 2.0 (±?), POD2 day 2.0 (±?), night 2.0 (±?)  2. POD0 day 2.7 (±?), night 3.0 (±?), POD1 day 3.3 (±?), night 3.0 (±?), POD2 day 2.0 (±?), night 2.0 (±?)  3. POD0 day 2.0 (±?), night 2.0 (±?), POD1 day 3.3 (±?), night 2.0 (±?), POD2 day 2.0 (±?), night 2.0 (±?) | |
| Allain 2019 (11) | 1. Sys  2. Loco  3. Loco  4. Loco | *Median VAS score 0-10 at rest.*  1. 0h 4.5 (2.0-7.0), 1h 4.0 (4.0-6.0), 2h 3.0 (2.0-4.0), 12h 3.0 (2.0-6.0), 24h 3.0 (2.0-6.0), 48h 2.0 (1.5-4.0)  2. 0h 5.0 (1.0-6.0), 1h 6.0 (1.0-6.0), 2h 4.0 (1.0-4.0), 12h 4.0 (2.0-5.0), 24h 3.0 (2.0-4.0), 48h 2.0 (2.0-4.0)  3. 0h 4.5 (0.0-6.0), 1h 3.0 (0.0-6.0), 2h 1.0 (0.0-3.0), 12h 3.0 (2.0-5.5), 24h 2.0 (1.5-4.5), 48h 2.0 (0.0-4.0)  4. 0h 4.5 (0.5-7.0), 1h 5.5 (3.0-6.0), 2h 3.0 (1.0-3.5), 12h 3.0 (3.0-4.0), 24h 3.0 (3.0-4.0), 48h 3.0 (2.0-4.0) | |
| Li 2020 (20) | 1. Loco  2. TEA | *Mean VAS score 0-10 at rest*  1. 3h 0.9 (±0.9)^c^, 24h 1.4 (±0.5)  2. 3h 2.0 (±0.6), 24h 2.0 (±1.1) | |
| Jung 2019 (21) | 1. Sys  2. Loco | *VAS score (descriptives unknown)*  1. POD0 2.35, POD1 2.06, POD2 1.91  2. POD0 2.15, POD1 1.56, POD2 1.50 | |
| Fernandez 2005 (22) | 1. TEA  2. Loco | *Median verbal rating score 0-3*  1. POD1 0 [0-3]  2. POD1 0 [0-3] | |
| **Epidural versus locoregional analgesia** | | | |
| Ishikawa 2012 (23) | 1. TEA  2. Loco | *Mean VAS score 0-10.*  1. 0h 4.0 (±?), 4h 2.8 (±?), 8h 3.3 (±?), 12h 3.5 (±?), 16h 3.5 (±?), 20h 3.3 (±?)  2. 0h 2.9 (±?), 4h 3.2 (±?), 8h 4.3 (±?), 12h 4.0 (±?), 16h 3.5 (±?), 20h 3.2 (±?) | |
| Spaans 2023 (10) | 1. Loco  2. TEA | *Mean NRS score 0-10*  1. POD0 recovery 2.2, POD0 night 2.8, POD1 day 3.0, POD1 night 2.0, POD2 day 1.8, POD2 night 1.5  2. POD0 recovery 1.5, POD0 night 0.9, POD1 day 1.5, POD1 night 1.9, POD2 day 2.0, POD2 night 1.9 | *Median proportion NRS scores ≥4 from POD0-3*  1. 11.1% (0.0-27.0)  2. 14.3% (0.0-33.0) |
| **Studies evaluating single analgesic techniques** | | | |
| **Systemic analgesia** | | | |
| Chen 2006 (25) | 1. Sys  2. Sys | *Mean VAS (95%CI) score 0-10*  1. POD1 6.9 (6.6-7.2)^c^, POD2 4.7 (4.4-5.0)  2. POD1 5.5 (5.2-5.9), POD2 4.4 (4.2-4.6) | |
| Freixinet 2004 (26) | 1. Sys | *Mean VAS score 1-10 before administering painkillers*  POD1 3.8 (±1.8), POD2 2.9 (±1.7) | |
| Zhong 2024 (27) | 1. Sys  2. Sys | *Median VAS score 0-10*  1. POD1 4.0 (1.0), POD2 2.0 (1.0)  2. POD1 4.0 (1.0), POD2 2.0 (1.0) | |
| Jeon 2016 (28) | 1. Sys  2. Sys | *Mean VAS score 0-10*  1. 0h 5.2 (±1.5), 8h 5.2 (±1.5), 16h 4.3 (±1.8), 24h 3.5 (±1.6)  2. 0h 6.5 (±2.3), 8h 5.3 (±2.2), 16h 4.5 (±2.0), 24h 4.2 (±2.1) | |
| Yamaguchi 2021 (29) | 1. Sys  2. Sys | *Mean NRS score 0-10*  1. POD0 5.3 (±?), POD1 3.9 (±?), POD2 3.5 (±?)  2. POD0 5.9 (±?), POD1 4.9 (±?), POD2 4.2 (±?) | |
| Kawaguchi 2021 (30) | 1. Sys  2. Sys | *NRS score 0-10 (descriptives unknown)*  1. Wound pain: ½h 6.5, 4h 4.9. POD1 3.9 and chest tube pain: ½h 5.8, 4h 4.9, POD1 4.0  2. Wound pain: ½h 5.0, 4h 2.9^c^, POD1 1.9^c^ and chest tube pain: ½h 1.0^c^, 4h 0.1^c^, POD1 0.1^c^ | |
| Horio 2002 (31) | 1. Sys  2. Sys | *Mean VAS 0-10*  1. POD1 1.2 (±0.9)  2. POD1 1.1 (±0.9) | |
| Kagimoto 2024 (32) | 1. Sys  2. Sys | *Mean NRS score 0-10*  1. Before tube removal: 4.4 (±2.5) and after tube removal: 1.6 (±1.0)  2. Before tube removal: 2.1 (±1.8)^c^ and after tube removal: 0.5 (±0.9)^c^ | |
| Rena 2008 (33) | 1. Sys  2. Sys | *Mean VAS score 0-10*  1. POD0 3.0 (±1.0), POD1 2.4 (±1.0), POD2 2.0 (±0.9)  2. POD0 3.2 (±0.9), POD1 2.5 (±1.0), POD2 2.0 (±0.9) | |
| Chen 2012 (34) | 1. Sys  2. Sys | *Mean VAS score 0-10 (range)*  1. POD1 4.7 (1.0-10.0), POD2 3.8 (1.0-9.0), POD3 3.0 (1.0-7.0)  2. POD1 5.7 (3.0-10.0)^c^, POD2 4.3 (2.0-8.0), POD3 3.4 (2.0-7.0) | |
| Wang 2016 (35) | 1. Sys  2. Sys  3. Sys | *Mean NRS score 0-10*  1. 1h 1.8 (±1.7), 8h 0.5 (±0.8), 16h 1.1 (±0.8), 24h 1.2 (±0.7), 32h 0.3 (±0.5), 40h 0.6 (±0.5), 48h 1.3 (±0.5), mean over 24h 0.9 (±0.3), max 24h 1.6 (±0.5)  2. 1h 3.0 (±1.7), 8h 1.6 (±2.0), 16h 2.2 (±2.4), 24h 1.6 (±1.0), 32h 0.6 (±1.2), 40h 1.3 (±1.6), 48h 1.1 (±0.9), mean over 24h 1.8 (±1.0), max 24h 3.5 (±1.9)  3. 1h 4.7 (±2.2)^c^, 8h 4.8 (±2.0)^c^, 16h 1.3 (±1.9), 24h 2.3 (±0.8)^c^, 32h 2.1 (±1.0), 40h 0.6 (±1.0), 48h 1.4 (±1.0), mean over 24h 2.8 (±1.0)^c^, max 24h 3.1 (±0.9)^c^ | |
| Masmoudi 2017 (36) | 1. Sys | *Mean VAS score 1-10*  48h 2.0 (±1.7) | |
| Hsu 2021 (37) | 1. Sys  2. Sys | *Mean VAS score (range unknown)*  1. POD1 2.9 (±2.0), POD2 2.4 (±1.8), POD3 1.7 (±1.3)  2. POD1 2.6 (±1.8), POD2 2.0 (±1.7), POD3 1.5 (±1.2) | |
| Kutluk 2018 (38) | 1. Sys  2. Sys  3. Sys | *Mean VAS score (range unknown)*  1. 4h 4.7 (±0.2), 24h 2.3 (±0.2)  2. 4h 6.1 (±0.3), 24h 3.1 (±0.2)  3. 4h 6.0 (±0.2)^c^, 24h 3.4 (±0.2)^c^ | |
| **Locoregional analgesia** | | | |
| Kim 2021 (24) | 1. Loco  2. Loco | *Mean NRS score 0-10 at rest.*  1. 3h 3.9 (±1.7), 6h 3.4 (±1.5), 12h 3.4 (±2.0)  2. 3h 4.0 (±1.8), 6h 4.0 (±1.8), 12h 3.3 (±1.7) | |
| Takamori 2024 (39) | 1. Loco | *Median (IQR) NRS scores 0-10*  1. On return to bed 2.0 (0.0-4.0), POD1 3.0 (1.0-4.0) | |
| Kiriyama 2024 (40) | 1. Loco  2. Loco | *Mean NRS score (range unknown)*  1. POD0 3h 4.0 (±?), 6h 3.9 (±?), POD1 3.3 (±?)  2. POD0 3h 3.1 (±?), 6h -, POD1 - | |
| Nachira 2018 (41) | 1. Loco  2. Loco | *Mean VAS score (range unknown)*  1. POD1 3.5 (±1.4)  2. POD1 6.4 (±2.5)^c^ | |
| Hwang 2018 (42) | 1. Loco  2. Loco | *Mean VAS score 0-10*  1. 1h 4.4 (±2.4)^c^, 24h 2.6 (±2.1)  2. 1h 1.4 (±1.4), 24h 1.6 (±1.3) | |
| Tsuboshima 2016 (43) | 1. Loco | *Mean NRS score (range unknown)*  6h 2.3 (±2.0), 24h 2.2 (±1.6), 48h 1.0 (±1.0) | |
| Hyland 2001 (44) | 1. Loco | *Mean pain score 0-3, immediately after surgery*  1. POD0 3 | |

*RCTs regarding analgesic techniques, ^b^ presented in mean ± sd or mean [range] or mean (95%-Confidence interval (CI)) or numbers with ^d^ are presented in median (low interquartile range (IQR) – high IQR) or median (IQR); ^c^ significant difference between groups within studies; Loco: locoregional, Sys: systemic, TEA: thoracic epidural analgesia, NRS: numeric pain rating score, VAS: visual analgesic score, POD: postoperative day, h: hour
