# Supplementary material for: Postoperative pain management after VATS for spontaneous pneumothorax - a systematic review
Source: BMC Anesthesiol. 2026 May 5;26:377. doi: 10.1186/s12871-026-03865-1 (PMC13289495; doi:10.1186/s12871-026-03865-1)
Supplement: Supplementary file 5 — Additional file 5. Surgical procedure, analgesic technique and multimodal medications per included article. [file 12871_2026_3865_MOESM5_ESM.docx]

# **Additional file 5. Surgical procedure, analgesic technique and multimodal medications per included article**

| Study | n | Block | Surgical procedure | Additional details anesthetic technique | Type and dose of systemic analgesia |
| --- | --- | --- | --- | --- | --- |
| **Regional versus systemic analgesia** | | | | | |
| Pompeo 2007 (17) | 1. 21  2. 22 | 1. TEA  2. Sys | 3port VATS bullectomy and abrasion | 1. Awake surgery. Removal at POD2. When insufficient pain control additional bolus via TEA  2. - | - |
| Liu 2022 (18) | 1. 162  2. 163 | 1. Loco  2. Sys | uVATS bullectomy | 1. Peroperative spontaneous ventilation  2. Peroperative mechanical ventilation | PCA morphine 1mg/ml. No difference in morphine use |
| Xie 2020 (19) | 1. 23  2. 34  3. 9 | 1. TEA  2. Loco  3. Sys | VATS mechanical pleurodesis | - | pcm iv/po, diazepam iv/po, and ketorolac iv. Rescue: iv/po opioids or opioid PCA. Opioid use lower in TEA group on POD0-1. No difference in PCA use. |
| Allain 2019 (11) | 1. 15  2. 9  3. 19  4. 16 | 1. Sys  2. Loco  3. Loco  4. Loco | uVATS bullectomy and chemical pleurodesis | - | pcm 1g qid, nefopam 20 mg qid, tramadol 50 mg qid. Rescue: 10mg oral rapid release morphine (when VAS>3). No difference in overall morphine consumption, but in group 1 more high-dose morphine (>50 mg) during first 72 hours. |
| Li 2020 (20) | 1. 34  2. 34 | 1. Loco  2. TEA | 2port VATS bullectomy with chemical pleurodesis | - | Parecoxib sodium 40mg/12h iv, morphine 10mg sc (VAS >4) |
| Jung 2019 (21) | 1. 52  2. 52 | 1. Sys  2. Loco | uVATS bullectomy | - | 1. –  2. Remifentanil 0.1-0.3mg iv |
| Fernandez 2005 (22) | 1. 22  2. 96 | 1. TEA  2. Loco | VATS bullectomy with apical parietal pleurectomy | 1. fentanyl 5 ug/ml and bupivacaine 0.1%, started prior to skin incision  2. occasionally in combination with ICNB or PVB | - |
| **Epidural versus locoregional analgesia** | | | | | |
| Ishikawa 2012 (23) | 1. 20  2. 20 | 1. TEA  2. Loco | 2port VATS bullectomy | 1. Removal of TEA when chest tube removal  2. Intrapleural analgesia through the chest tube | Diclofenac 25 mg. No difference in use |
| Spaans 2023 (10) | 1. 96  2. 122 | 1. Loco  2. TEA | Uni- or multiportal VATS bullectomy with pleurectomy | 1. -  2. removal on POD2-3 | pcm, nsaid, opioids. No difference in mean opioid dose, but the proportion of patients in group 1 requiring opioids was higher on POD0-1. |
| **Studies evaluating single analgesic techniques** | | | | | |
| **Systemic analgesia** | | | | | |
| Chen 2006 (25) | 1. 103  2. 99 | 1. Sys  2. Sys | 1. VATS bullectomy and abrasion with chemical pleurodesis via chest tube  2. VATS bullectomy and abrasion | - | 1. pcm, nsaid, Demerol 50mg/ampule (VAS>7), mean accumulated dose 115 mg^a^  2. pcm, nsaid, Demerol 50mg/ampule (VAS>7), mean accumulated dose 69 mg |
| Freixinet 2004 (26) | 1. 46 | 1. Sys | 2port VATS bullectomy and abrasion | - | 1. Metamizol 2g/6h until POD2, then 0.6 g/6h until POD7, buprenorphine 0.3 mg i.m. (VAS>7). Extra doses necessary mean 0.9 (±2.9) |
| Zhong 2024 (27) | 1. 65  2. 63 | 1. Sys  2. Sys | 1. VATS bullectomy with abrasion  2. VATS bullectomy with chemical pleurodesis | - | Loxoprofen 60 mg bid |
| Jeon 2016 (28) | 1. 40  2. 46 | 1. Sys  2. Sys | 1. uVATS bullectomy with abrasion  2. Multiportal VATS bullectomy with abrasion | - | Nsaid and PCA with fentanyl |
| Yamaguchi 2021 (29) | 1. 23  2. 50 | 1. Sys  2. Sys | 3port VATS bullectomy with rehabilitation  2. 3port VATS bullectomy | - | pcm iv, loxoprofen 180 mg/day and PCA 1mg fentanyl in normal saline to 0.005 mg/kg/h until POD1. Rescue: PCA and diclofenac 25mg |
| Kawaguchi 2021 (30) | 1. 21  2. 21 | 1. Sys  2. Sys | 1. 3port VATS bullectomy  2. 3port VATS bullectomy with early chest tube removal | - | 24hours routine roxoprofen tid. Rescue: 50mg flurbiprofen axetil, 15mg pentazocine, or 1000-mg pcm iv every 6h |
| Horio 2002 (31) | 1. 53  2. 50 | 1. Sys  2. Sys | 1. 3port VATS bullectomy and abrasion  2. 3port VATS bullectomy | - | 1. Indomethacin 50 mg/tablet, accumulated requirement of tablet mean 0.2 (±0.7)  2. Indomethacin 50 mg/tablet, accumulated requirement of tablet mean 0.2 (±0.6) |
| Kagimoto 2024 (32) | 1. 22  2. 21 | 1. Sys  2. Sys | 1. 3port VATS bullectomy with long chest tube length  2. 3port VATS bullectomy with short chest tube length | - | Routine loxoprofen 60 mg tid. Rescue: flurbiprofen iv, pcm, diclofenac, pentazocine im or tramadol |
| Rena 2008 (33) | 1. 112  2. 108 | 1. Sys  2. Sys | 1. 3port VATS bullectomy with abrasion  2. 3port VATS bullectomy with pleurectomy | - | 1.2mg/kg/h pcm and continuous 0.15 mg/kg/h tramadol iv |
| Chen 2012 (34) | 1. 80  2. 80 | 1. Sys  2. Sys | 1. 3port VATS bullectomy with pleurectomy  2. 3port VATS bullectomy with abrasion and minocycline | - | 1. pcm, nsaid. Rescue: meperidine i.m. 50mg/ampule every 4-6h (VAS>7). Mean accumulated dose 43.1 (±81.8) mg  2. Acetaminophen, nsaid. Rescue: meperidine i.m. 50mg/ampule every 4-6h (VAS>7). Mean accumulated dose 61.9 (±74.3)mg^c^ |
| Wang 2016 (35) | 1. 14  2. 26  3. 17 | 1. Sys  2. Sys  3. Sys | 1. subxiphoid VATS bullectomy with abrasion  2. uVATS bullectomy with abrasion  3. 2port VATS bullectomy with abrasion | - | Diclofenac 25mg qid, morphine 0.1mg/kg i.v. (VAS>4) |
| Masmoudi 2017 (36) | 1. 351 | 1. Sys | uVATS bullectomy with mechanical or chemical pleurodesis | - | pcm 1000mg qid, nefopam 20mg 4-5 times a day, tramadol 100mg tid. Rescue: PCA morphine 1mg/ml. First 48h mean cumulative quantity morphine 37.2 (±26.1) mg |
| Hsu 2021 (37) | 1. 102  2. 102 | 1. Sys  2. Sys | 1. 3port VATS bullectomy with abrasion with mesh  2. 3port VATS bullectomy with abrasion | - | pcm, nsaid, meperidine 50mg/ampule every 4-6h (VAS>7) |
| Kutluk 2018 (38) | 1. 45  2. 45  3. 45 | 1. Sys  2. Sys  3. Sys | 1. uVATS bullectomy with pleurectomy  2. 2port VATS bullectomy with pleurectomy  3. 3port VATS bullectomy with pleurectomy | - | pcm 1g/6h, tenoxicam 20mg/12h, Rescue: tenoxicam im |
| **Locoregional analgesia** | | | | | |
| Kim 2021 (24) | 1. 25  2. 25 | 1. Loco  2. Loco | uVATS bullectomy | 1. Block placed at both sides of incision  2. - | pcm 650mg tid, ketorolac 30 mg iv (NRS 4-5) or fentanyl 50 ug iv (NRS >6). Group 1 significantly more use of ketorolac. |
| Takamori 2024 (39) | 1. 54 | 1. Loco | uVATS bullectomy | - | - |
| Kiriyama 2024 (40) | 1. 50  2. 24 | 1. Loco  2. Loco | 1. 3port VATS bullectomy and glucose intrapleural spray  2. 3port VATS bullectomy | - | - |
| Nachira 2018 (41) | 1. 23  2. 23 | 1. Loco  2. Loco | 1. uVATS bullectomy with chemical or mechanical pleurodesis  2. 3port VATS bullectomy with chemical or mechanical pleurodesis | - | - |
| Hwang 2018 (42) | 1. 20  2. 21 | 1. Loco  2. Loco | 1. VATS bullectomy with intubation  2. VATS bullectomy without intubation | - | Diclofenac 30 mg |
| Tsuboshima 2016 (43) | 1. 25 | 1. Loco | uVATS bullectomy | - | Additional PCA necessary in 4% of the patients |
| Hyland 2001 (44) | 1. 22 | 1. Loco | 3port VATS bullectomy with apical pleurectomy | ss-ICNB with PCA or continuous ICNB without PCA | Additional PCA in case of ss-ICNB |

*RCTs regarding analgesic techniques; n: number of patients, (u)VATS: (uniportal) video-assisted thoracic surgery, Loco: locoregional, Sys: systemic, TEA: thoracic epidural analgesia, PCA: patient controlled analgesia, PCM: paracetamol or acetaminophen, NSAID: non-steroidal anti-inflammatory drugs, h: hour, qid: four times a day, tid: three times a day, bid: twice a day, NRS: numeric pain rating score, VAS: visual analgesic score, POD: postoperative day, sc: subcutaneous, iv: intravenous, im: intramuscular, ±: standard deviation. If doses of analgesia are not reported, it is not presented by the article.
